# Mesenchymal stem cells added to second-line therapy improve response and failure-free survival in steroid-refractory acute graft-versus-host disease after allogeneic hematopoietic stem cell transplantation: A meta-analysis of randomized controlled trials

**DOI:** 10.3389/fonc.2025.1704963

**Published:** 2025-11-04

**Authors:** Man Xiao, Yinghu Yan, Rui Zhang, Dan Wang, Li Wang, Yameng Zhao, Jinhong Yang

**Affiliations:** ^1^ Nursing School, Shandong Second Medical University, Weifang, Shandong, China; ^2^ Department of Oncology, Sunshine Union Hospital, Weifang, Shandong, China; ^3^ Department of Anesthesiology, Rocket Force Characteristic Medical Center, Beijing, China; ^4^ Department of Emergency Medicine, Rocket Force Characteristic Medical Center, Beijing, China; ^5^ Nursing Department, Sunshine Union Hospital, Weifang, Shandong, China; ^6^ Department of Respiratory Medicine, Rocket Force Characteristic Medical Center, Beijing, China

**Keywords:** mesenchymal stem cells, steroid-refractory, acute graft-versus-host disease, hematopoietic stem cell transplantation, meta-analysis, randomized controlled trial

## Abstract

**Background:**

Steroid-refractory acute graft-versus-host disease (aGVHD) remains a major cause of morbidity and mortality after allogeneic hematopoietic stem cell transplantation (allo-HSCT), with limited effective treatment options. Mesenchymal stem cells (MSCs) have emerged as a promising therapeutic approach due to their immunomodulatory and tissue-repair properties. However, inconsistent results existed.

**Methods:**

A systematic literature search was conducted in PubMed, Embase, and the Cochrane Central Register of Controlled Trials up to May 2025. Randomized controlled trials (RCTs) evaluating MSCs plus second-line therapy versus second-line therapy alone in patients with steroid-refractory aGVHD were included. Meta-analysis was performed using random-effects models to pool risk ratios (RR) or hazard ratios (HR) with 95% confidence intervals (CIs).

**Results:**

Four RCTs comprising 650 patients were included. MSC administration significantly improved overall response (RR: 1.13, 95% CI: 1.03-1.23, P = 0.007) and complete response rates (RR: 1.43, 95% CI: 1.19-1.70, P < 0.001). Subgroup analysis showed consistent benefits in patients with skin or gut involvement, multiorgan disease, and adults. MSC treatment also reduced the incidence of chronic GVHD (HR: 0.60, 95% CI: 0.42-0.86, P = 0.005) and improved failure-free survival (HR: 0.72, 95% CI: 0.54-0.95, P = 0.022), although no significant overall survival benefit was observed. The safety profile was comparable with controls.

**Conclusions:**

The addition of MSCs to second-line therapy significantly improves treatment response, reduces chronic GVHD incidence, and prolongs failure-free survival in patients with steroid-refractory aGVHD, with a favorable safety profile. These findings support MSC-based therapy as a promising strategy for this high-risk population.

## 1 Introduction

Allogeneic hematopoietic stem cell transplantation (allo-HSCT) is a curative treatment for most hematological malignancies (1). However, 40% to 60% of recipients develop moderate to severe acute graft-versus-host disease (aGVHD) (2, 3), which remains one of the most common complications and a leading cause of mortality following allo-HSCT (4).

Corticosteroids represent the standard first-line therapy for aGVHD (5, 6), yet complete response is achieved in only approximately 50% of patients (7, 8). Response rates are particularly poor among those with visceral and/or multiorgan involvement (9). To date, no optimal second-line treatment has been established for steroid-refractory aGVHD, and the prognosis for these patients remains dismal (4). Hence, there is an urgent need for more effective therapeutic strategies.

Mesenchymal stem cells (MSCs) exhibit broad immunomodulatory capacity through their ability to regulate key immune populations—including T and B lymphocytes, natural killer cells, and dendritic cells—by influencing their proliferation, activation, and maturation (10–13). Furthermore, MSCs promote the expansion of regulatory T cells and help rebalance the Th1/Th2 ratio (14). Coupled with their multipotent differentiation potential toward mesodermal lineages, these immunoregulatory and regenerative properties provide a strong mechanistic rationale for exploring MSC-based therapies in aGVHD.

Several clinical studies have explored MSCs specifically for steroid-refractory aGVHD (15–18). However, reported outcomes regarding their efficacy remain inconsistent, and their definitive role in managing this condition continues to be debated. Given that individual trials are often limited by small sample sizes and inadequate statistical power to definitively establish clinical benefits, we performed a meta-analysis of randomized controlled trials (RCTs) to comprehensively evaluate the efficacy and safety of MSCs combined with second-line therapy in patients with steroid-refractory aGVHD after allo-HSCT.

## 2 Materials and methods

### 2.1 Search strategy

A comprehensive systematic search was performed in PubMed, Embase (Excerpta Medica Database), and the Cochrane Central Register of Controlled Trials (CENTRAL) using keywords and Medical Subject Headings (MeSH) terms related to “mesenchymal stem cells,” “mesenchymal stromal cells,” “MSC,” “graft versus host disease,” “GVHD,” and “graft vs. host disease”. The search aimed to identify RCTs evaluating the efficacy and safety of MSCs in patients with steroid-refractory acute GVHD following allo-HSCT for hematological diseases.

The search included all publications available up to May 2025 without language restrictions. Detailed search strategies for each database are provided in **Supplementary Tables 1-3**. Additionally, the reference lists of all included studies were manually screened to identify further relevant publications. This meta-analysis was designed and reported in accordance with the Preferred Reporting Items for Systematic Reviews and Meta-Analyses (PRISMA) guidelines (19).

### 2.2 Selection criteria

Two investigators independently assessed all potentially eligible studies. RCTs evaluating the efficacy and safety of MSCs in patients with steroid-refractory aGVHD following allo-HSCT for hematological diseases were included. Eligibility was independent of patient demographics, donor-recipient human leukocyte antigen (HLA) matching, graft source, conditioning regimens, GVHD prophylaxis, and MSC origin, dosage, or timing of administration.

Steroid-refractory aGVHD was defined as progression within 3 days, no improvement after 7 days, failure to achieve complete response by day 14 of steroid therapy, or recurrence during steroid taper.

The primary endpoint was the overall response (OR) rate at day 28, comprising complete response (CR) or partial response (PR). CR was defined as the complete resolution of manifestations in all affected organs. PR referred to improvement of at least one stage in every initially involved organ, without progression in any other organ. Secondary efficacy endpoints included cumulative incidence of chronic GVHD, failure-free survival (FFS), and overall survival (OS). FFS was defined as the time from randomization to the earliest event among relapse or progression of the underlying disease, non-relapse mortality, or initiation of new systemic aGVHD therapy. OS referred to the time from randomization to death from any cause. Safety endpoints encompassed adverse events (AEs) and severe AEs graded according to the National Cancer Institute Common Terminology Criteria for Adverse Events (CTCAE), as well as rates of disease relapse and infections.

Any discrepancies between the two investigators were resolved through discussion or by consulting a third specialist.

### 2.3 Data extraction

Two investigators independently extracted data from the included studies. The following information was collected: first author, year of publication, patient characteristics (underlying diagnosis and sample size), donor type (related or unrelated), HLA compatibility (matched, mismatched, or haploidentical), source of hematopoietic stem cells (bone marrow, peripheral blood, or umbilical cord blood), conditioning regimen (busulfan-based or total body irradiation-based), GVHD prophylaxis, MSC origin (umbilical cord [UC], bone marrow [BM], or adipose tissue), and MSC dosage, frequency of administration, and all prespecified outcomes.

Any discrepancies between the two investigators were resolved through consensus or by consulting a third expert. When necessary, the corresponding authors of the original studies were contacted to obtain missing or incomplete data.

### 2.4 Methodological quality evaluation

The methodological quality of the included studies was evaluated using the Cochrane Risk of Bias Tool, as recommended in the Cochrane Handbook for Systematic Reviews of Interventions (Version 5.1.0) (20). Two investigators independently assessed each study and assigned ratings of “high,” “low,” or “unclear” risk of bias across the following seven domains: random sequence generation, allocation concealment, blinding of participants and personnel, blinding of outcome assessment, incomplete outcome data, selective reporting, and other potential sources of bias. Any discrepancies between the investigators were resolved through discussion.

### 2.5 Statistical analyses

Risk ratios (RR) with 95% confidence intervals (CI) were calculated for dichotomous outcomes. For time-to-event data, hazard ratios (HR) and corresponding 95% CIs were pooled. Heterogeneity among studies was evaluated using the I² statistic, with I² > 50% and a P-value < 0.10 indicating substantial heterogeneity (21). A random-effects model was applied for all meta-analyses to yield more conservative estimates (22), regardless of heterogeneity levels. Predefined subgroup analyses were performed based on the following variables: aGVHD severity (grades II-IV vs. III-IV), organ involvement (e.g., liver, skin, gut), number of organs affected (single vs. multiple), patient age (children vs. adults), and MSC source (UC vs. BM). A P-value < 0.05 was considered statistically significant. All analyses were conducted using Stata version 12.0 (Stata Corporation, College Station, TX, USA).

## 3 Results

### 3.1 Study selection and characteristics

**Figure 1:**
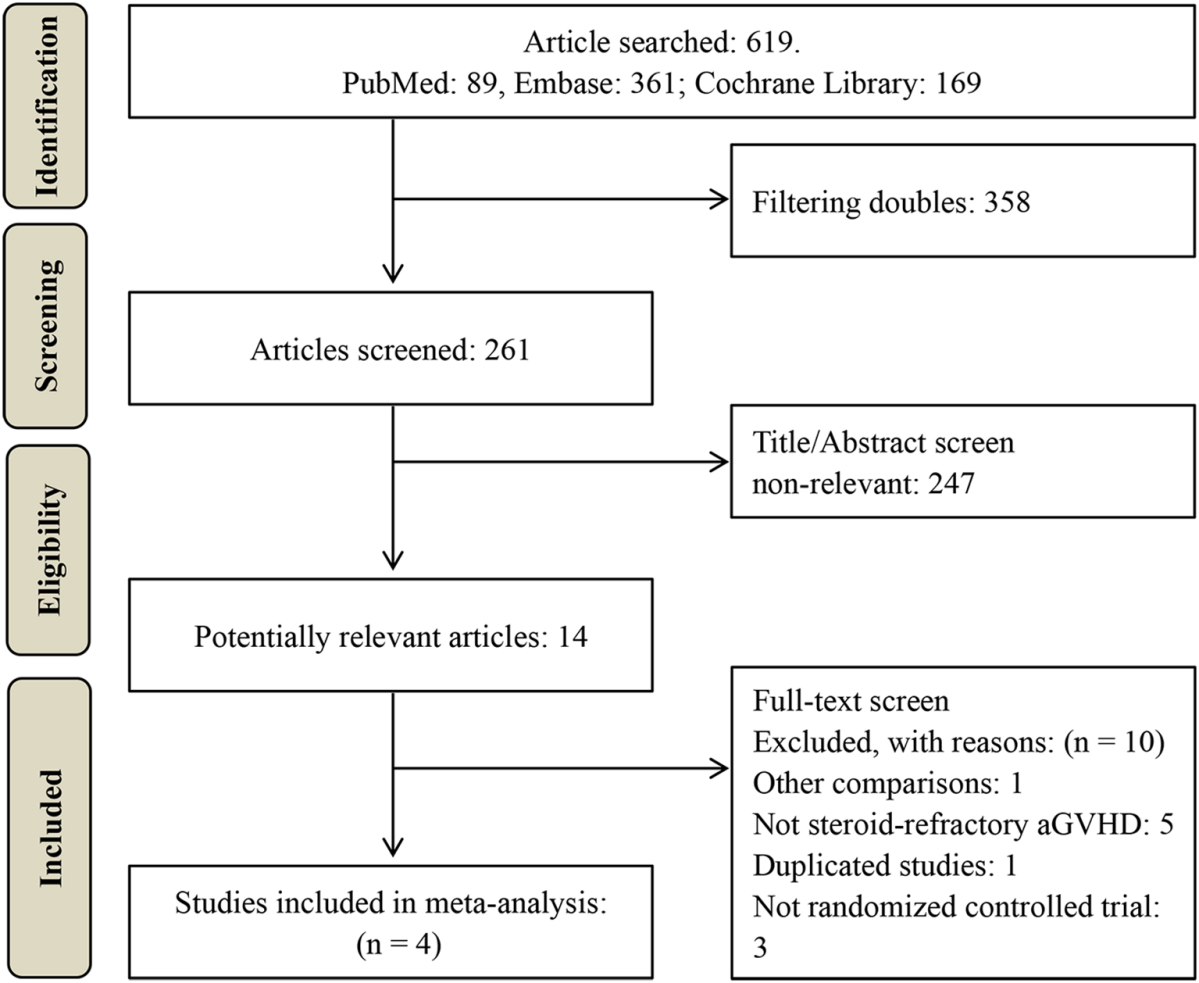
Study selection flow diagram.

A total of 619 potentially relevant studies were initially identified through the literature search. After removing duplicates, 261 records underwent title and abstract screening, resulting in the exclusion of 247 articles. The remaining 14 studies were subjected to full-text assessment, of which 10 were excluded. Ultimately, four RCTs comprising 650 patients were included in the meta-analysis (23–26), as illustrated in **Figure 1**. The characteristics of these studies are summarized in **Tables 1**, **2**.

**Table 1:** Characteristics for the included randomized controlled trials.

Studies	Published year	Study design	Phase	Sample sizes (n) MSC/control	GVHD grade	Control	MSC source	MSC dose and frequency	Study endpoints
Jiang et al. (23)	2024	Multicenter RCT	II	40/38	SR-aGVHD grade II-IV	Standardized second-line treatment (except for ruxolitinib) + placebo	UC	1.0×10^6^ cells/kg twice weekly for 4 consecutive weeks for patients who achieved CR or 8 consecutive weeks for patients who achieved PR.	Primary endpoint: OR rate at day 28; secondary endpoints: chronic GVHD, FFS, OS
Fu et al. (24)	2024	Multicenter RCT	III	65/65	SR-aGVHD grade II-IV	Basiliximab	UC	1.0×10^6^ cells/kg once weekly for 4 consecutive weeks for patients who achieved CR or 8 consecutive weeks for patients who achieved PR.	Primary endpoint: CR rate at day 28; secondary endpoints: PR rate at day 28, chronic GVHD, FFS, OS
Zhao et al. (25)	2022	Multicenter RCT	III	101/102	SR-aGVHD grade II-IV	Basiliximab + calcineurin inhibitor	BM	1.0×10^6^ cells/kg once weekly for 4 consecutive weeks for patients who achieved CR or 8 consecutive weeks for patients who achieved PR.	Primary endpoint: OR rate at day 28; secondary endpoints: chronic GVHD, FFS, OS
Kebriaei et al. (26)	2020	Multicenter RCT	III	173/87	SR-aGVHD grade B to D	Standardized second-line treatment + placebo	BM	Remestemcel-L, 2.0×10^6^ cells/kg twice weekly for consecutive 4 weeks for patients who achieved CR, four additional infusions over 4 weeks for patients with incomplete response.	Primary endpoints: durable CR, OR rate at day 28; secondary endpoint: OS

GVHD, graft-versus-host disease; SR-aGVHD, steroid-refractory acute graft-versus-host disease; MSC, mesenchymal stem cell; RCT, randomized controlled trial; UC, umbilical cord; BM, bone marrow; CR, complete response; PR, partial response; OR, overall response; FFS, failure-free survival; OS, overall survival.

**Table 2:** Summary of the transplantation-related characteristics of the included randomized controlled trials.

Studies	Patients’ diagnosis	Donor characteristics, n (%)		HSC source, n (%)		Conditioning, n (%)		GVHD prophylaxis, n (%)	
		MSC group	Control group	MSC group	Control group	MSC group	Control group	MSC group	Control group
Jiang et al. (23)	Hematological malignancies	Sibling: 14 (35.0%), unrelated: 4 (10.0%), haploidentical: 22 (55.0%)	Sibling: 10 (26.3%), unrelated: 2 (5.3%), haploidentical: 26 (68.4%)	PBSC: 36 (90.0%), PBSC+BM: 4 (10.0%), CB: 0 (0%)	PBSC: 33 (86.8%), PBSC+BM: 4 (10.5%), CB: 1 (2.6%)	Bu-based: 33 (82.5%), TBI-based: 6 (15.0%), others: 1 (2.5%)	Bu-based: 31 (81.6%), TBI-based: 7 (18.4%), others: 0 (0%)	MTX + CNIs: 37 (92.5%), MTX excluded: 3 (7.5%)	MTX + CNIs: 34 (89.5%), MTX excluded: 4 (10.5%)
Fu et al. (24)	Hematological malignancies, SAA	Sibling: 1 (1.5%), unrelated: 1 (1.5%), haploidentical: 63 (96.9%)	Sibling: 3 (4.6%), unrelated: 0 (0%), haploidentical: 62 (95.4%)	PBSC: 59 (90.8%), PBSC+BM: 6 (9.2%)	PBSC: 54 (83.1%), PBSC+BM: 11 (16.9%)	Bu-based: 62 (95.4%), TBI-based: 3 (4.6%)	Bu-based: 62 (95.4%), TBI-based: 3 (4.6%)	CsA+MMF+MTX: 63 (96.9%), CsA+MMF+MTX+low-dose PTCy: 2 (3.1%)	CsA+MMF+MTX: 62 (95.4%), CsA+MMF+MTX+low-dose PTCy: 3 (4.6%)
Zhao et al. (25)	Hematological malignancies	HLA matched: 51 (51.5%), HLA mismatched: 48 (48.5%)	HLA matched: 51 (51.5%), HLA mismatched: 48 (48.5%)	PBSC: 53 (53.5%), PBSC+BM: 46 (46.5%)	PBSC: 57 (57.6%), PBSC+BM: 42 (42.4%)	Bu-based: 51 (51.5%), TBI-based: 48 (48.5%)	Bu-based: 42 (42.4%), TBI-based: 57 (57.6%)	CsA + MTX or CsA + MTX+ MMF: 42 (42.4%), CsA + MTX + MMF + ATG: 57 (57.6%)	CsA + MTX or CsA + MTX+ MMF: 43 (43.4%), CsA + MTX + MMF + ATG: 56 (56.6%)
Kebriaei et al. (26)	Hematological diseases	Unrelated: 93 (57%), related: 70 (43%)	Unrelated: 47 (58%), related: 34 (42%)	BM: 20 (12%), PBSC: 127 (78%), CB: 16 (10%)	BM: 14 (17%), PBSC: 57 (70%), CB: 10 (12%)	NA	NA	NA	NA

HSC, hematopoietic stem cell; GVHD, graft-versus-host disease; MSC, mesenchymal stem cell; HLA, human leukocyte antigen; SAA, severe aplastic anemia; PBSC, peripheral blood stem cell; BM, bone marrow; CB, cord blood; Bu-based, busulfan-based; TBI-based, total body irradiation-based; MTX: methotrexate; CNIs: calcineurin inhibitors; CsA: cyclosporine A; MMF: mycophenolate mofetil; PTCy: post-transplantation cyclophosphamide; ATG, anti-thymocyte globulin; NA, not available.

All included studies were multicenter RCTs, including one phase II trial (23) and three phase III trials (24–26). Sample sizes ranged from 78 to 260 participants. All patients exhibited steroid-refractory aGVHD of grade II or above. Control groups received institutional standard second-line therapy for steroid-refractory aGVHD, whereas the intervention groups received additional MSC treatment. The source of MSCs was BM in two studies (25, 26) and UC in the other two (23, 24). Dosing regimens varied: two studies administered MSCs at 1×10^6^ cells/kg once weekly for 4 weeks in patients achieving CR or 8 weeks in those with PR (24, 25); one study used a similar dose but administered twice weekly (23); and the remaining study administered 2×10^6^ cells/kg twice weekly (26). One study enrolled exclusively adult patients (24), whereas the other three included both pediatric and adult populations (23, 25, 26).

### 3.2 Methodological quality evaluation

All four included RCTs described the process of randomization and provided detailed methods for random sequence generation (23–26). Two of the trials were conducted in an open-label manner (24, 25), whereas the other two were double-blinded (23, 26). Blinding of outcome assessors was implemented in all studies (23–26). With regard to incomplete outcome data, selective reporting, and other potential sources of bias, all trials were judged to be at low risk. A summary of the methodological quality assessment is presented in **Supplementary Figure 1**.

### 3.3 Overall response, complete response, and subgroup analysis

**Figure 2:**
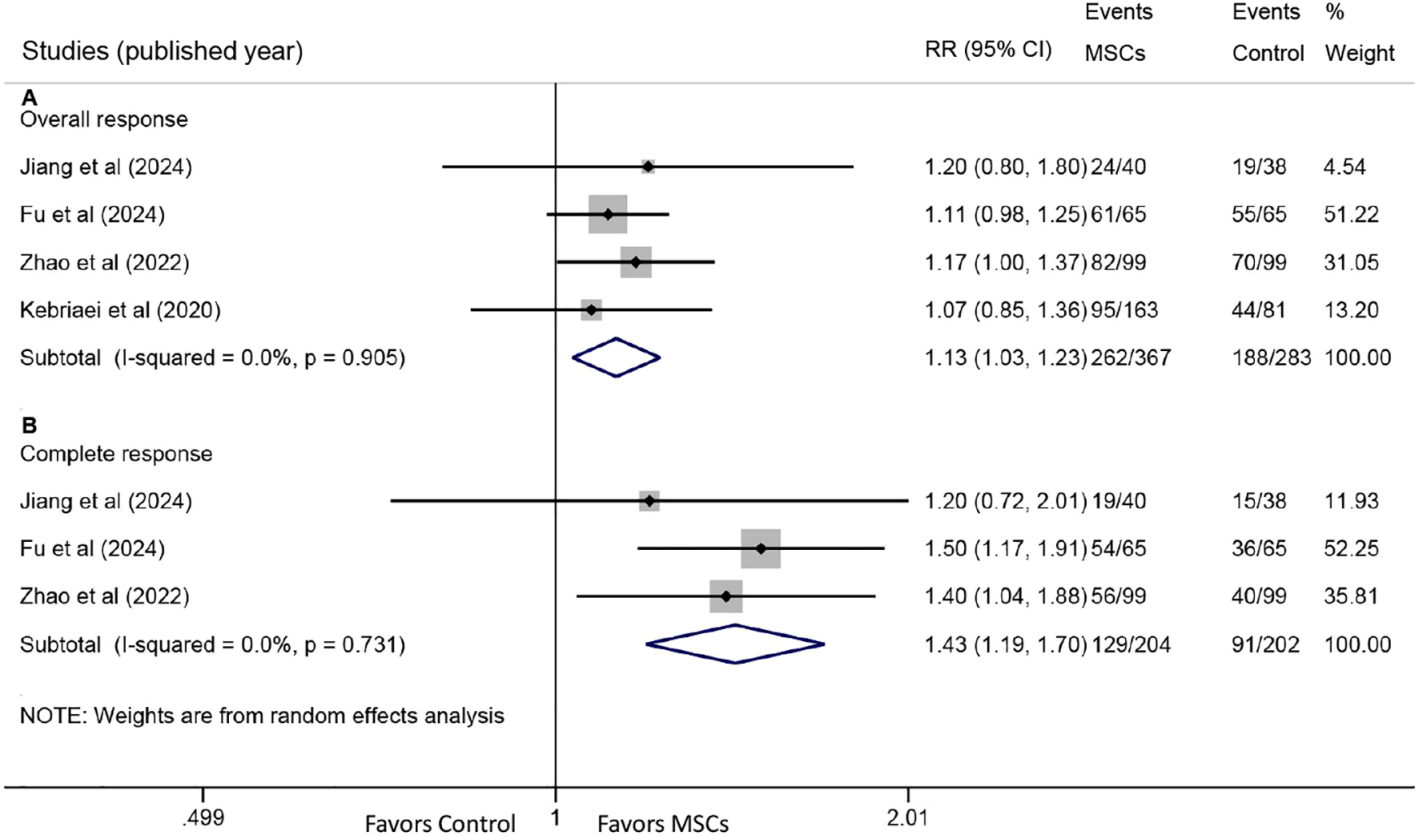
Forest plots for overall response (OR) and complete response (CR). **(A)** Forest plot comparing the OR rate at day 28 between the MSC group and the control group. **(B)** Forest plot comparing the CR rate at day 28 between the MSC group and the control group.

All four studies reported OR rates. Pooled analysis demonstrated a significantly higher OR rate in the MSC group compared with the control group (RR: 1.13, 95% CI: 1.03-1.23, P = 0.007) (**Figure 2A**). CR rates were reported in three studies, with pooled results also showing a significant improvement in the MSC group (RR: 1.43, 95% CI: 1.19-1.70, P < 0.001) (**Figure 2B**).

**Figure 3:**
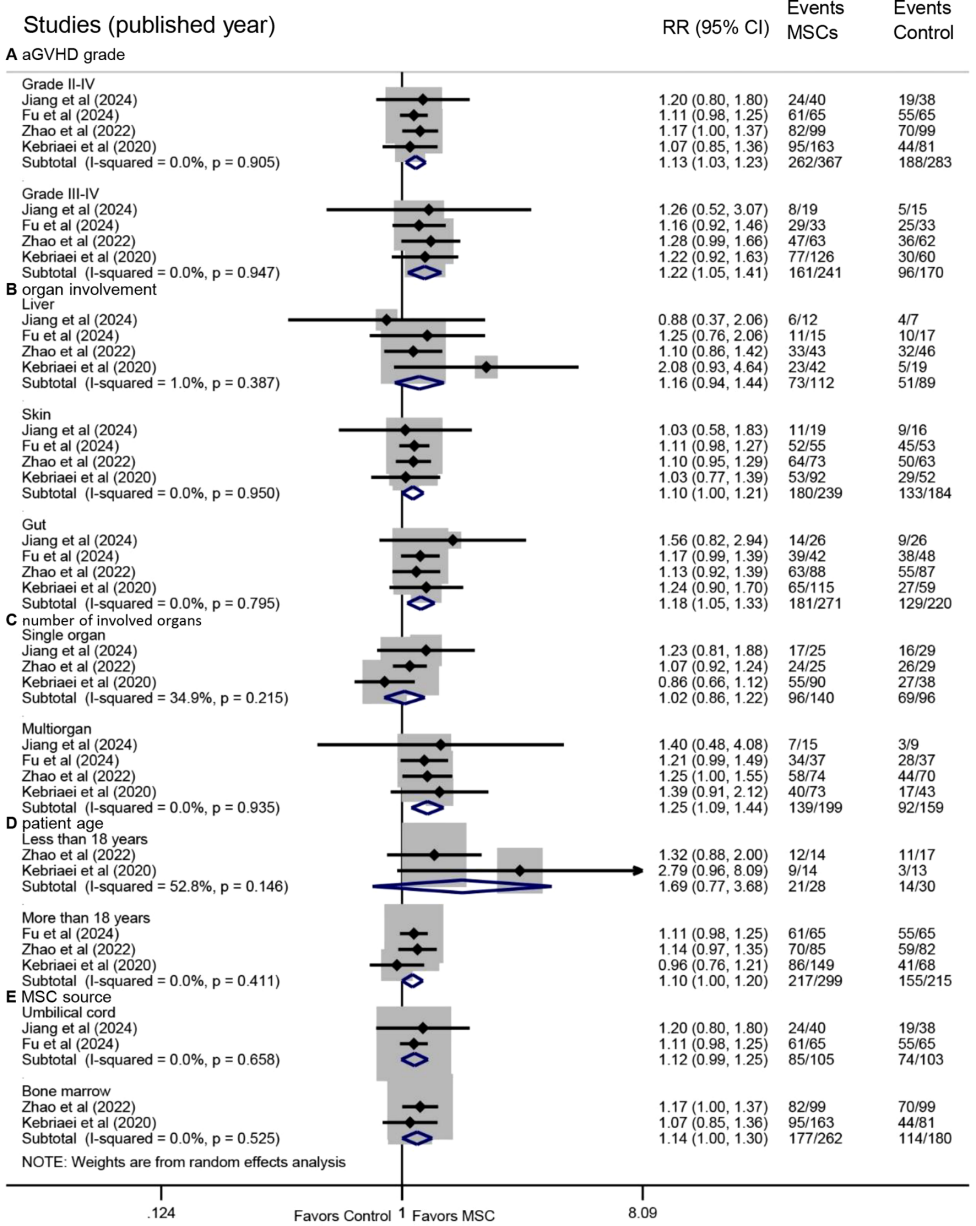
Forest plots of subgroup analyses for overall response. Subgroup analyses were performed based on **(A)** aGVHD grade, **(B)** organ involvement, **(C)** number of involved organs, **(D)** patient age, and **(E)** MSC source. aGVHD, acute graft-versus-host disease.

Prespecified subgroup analyses based on OR data revealed consistent benefits across several subgroups. Improved OR was observed in both grade II-IV (RR: 1.13, 95% CI: 1.03-1.23, P = 0.007) and grade III-IV aGVHD (RR: 1.22, 95% CI: 1.05-1.41, P = 0.008) (**Figure 3A**). Significant OR enhancement was identified in patients with skin involvement (RR: 1.10, 95% CI: 1.00-1.21, P = 0.044) and gut involvement (RR: 1.18, 95% CI: 1.05-1.33, P = 0.006), but not in those with liver involvement (RR: 1.16, 95% CI: 0.94-1.44, P = 0.167) (**Figure 3B**).

Multiorgan involvement was associated with a significantly higher OR following MSC treatment (RR: 1.25, 95% CI: 1.09-1.44, P = 0.002), whereas no significant benefit was observed in single-organ involvement (RR: 1.02, 95% CI: 0.86-1.22, P = 0.803) (**Figure 3C**). A statistically significant improvement was seen in adult patients (RR: 1.10, 95% CI: 1.00-1.20, P = 0.048), but not in pediatric patients (RR: 1.69, 95% CI: 0.77-3.68, P = 0.188) (**Figure 3D**). Furthermore, MSC administration derived from BM was associated with a significant improvement in OR (RR: 1.14, 95% CI: 1.00-1.30, P = 0.047), whereas those from UC showed a trend without reaching statistical significance (RR: 1.12, 95% CI: 0.99-1.25, P = 0.063) (**Figure 3E**).

### 3.4 Chronic GVHD, failure-free survival, and overall survival

**Figure 4:**
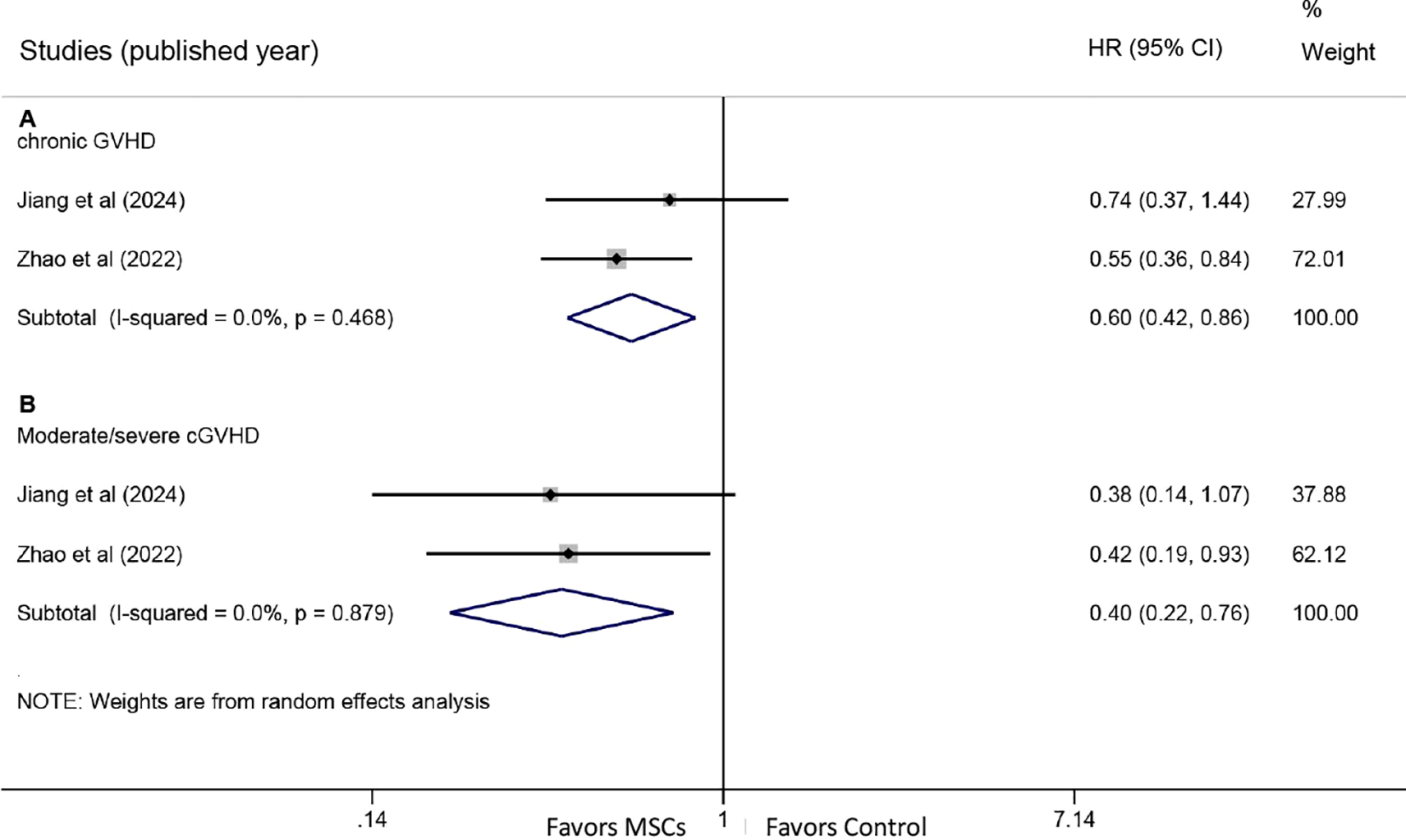
Forest plots for chronic graft-versus-host disease. **(A)** Forest plot comparing the cumulative incidence of all-grade chronic GVHD between the MSC group and the control group. **(B)** Forest plot comparing the incidence of moderate/severe chronic GVHD between the MSC group and the control group.

Two studies provided HR data for chronic GVHD. Pooled analysis demonstrated a significantly lower cumulative incidence of chronic GVHD in the MSC group compared with the control group (HR: 0.60, 95% CI: 0.42-0.86, P = 0.005) (**Figure 4A**). Additionally, the incidence of moderate/severe chronic GVHD was also significantly reduced in the MSC group (HR: 0.40, 95% CI: 0.22-0.76, P = 0.005) (**Figure 4B**).

**Figure 5:**
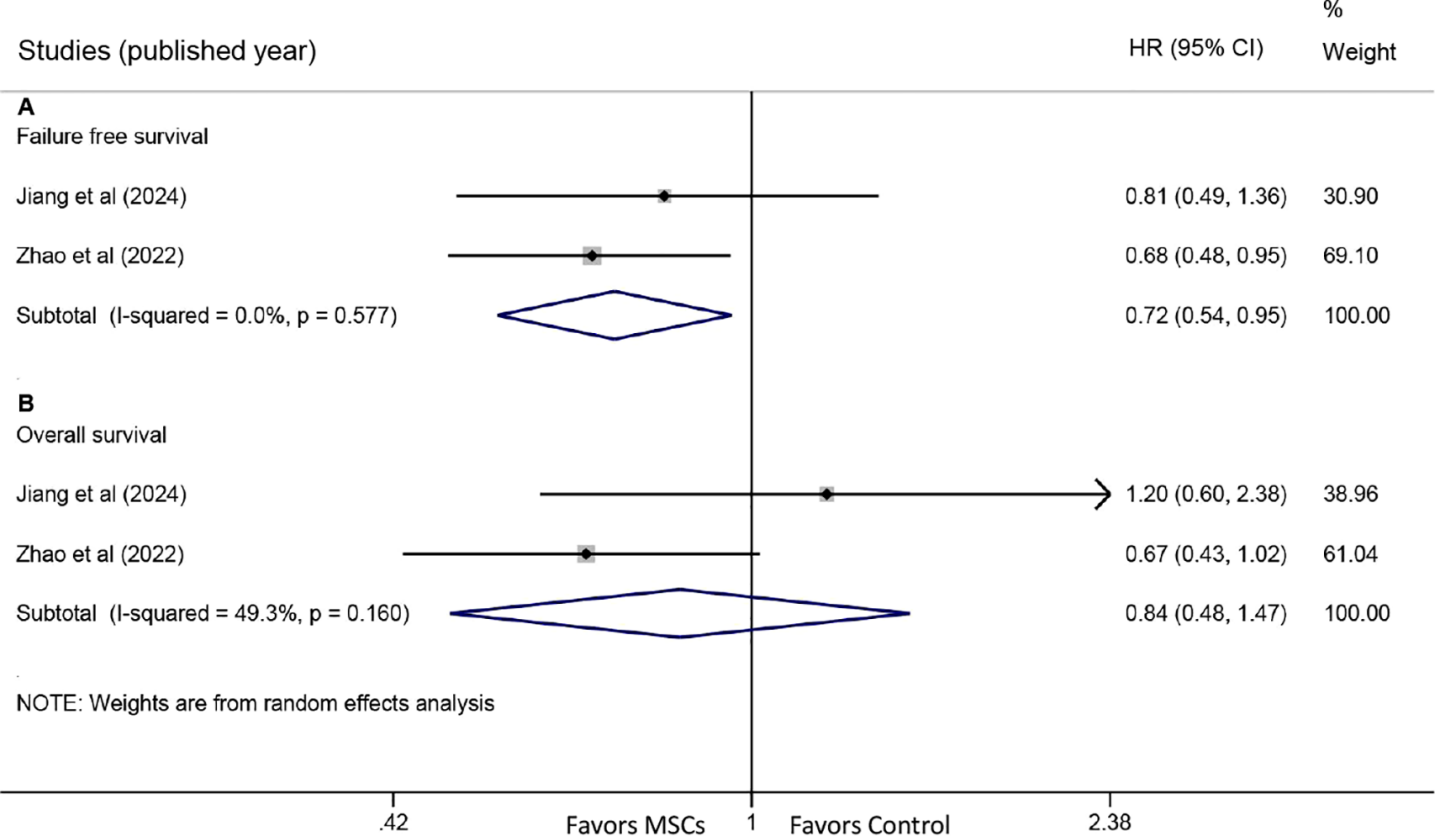
Forest plots for failure-free survival (FFS) and overall survival (OS). **(A)** Forest plot comparing FFS between the MSC group and the control group. **(B)** Forest plot comparing OS between the MSC group and the control group.

Two studies reported HRs for FFS. The meta-analysis revealed a significant improvement in FFS with MSC treatment (HR: 0.72, 95% CI: 0.54-0.95, P = 0.022) (**Figure 5A**). Similarly, two studies provided HR data for OS. Pooled results indicated no statistically significant difference in OS between the MSC and control groups (HR: 0.84, 95% CI: 0.48-1.47, P = 0.542) (**Figure 5B**).

### 3.5 Safety profile

**Figure 6:**
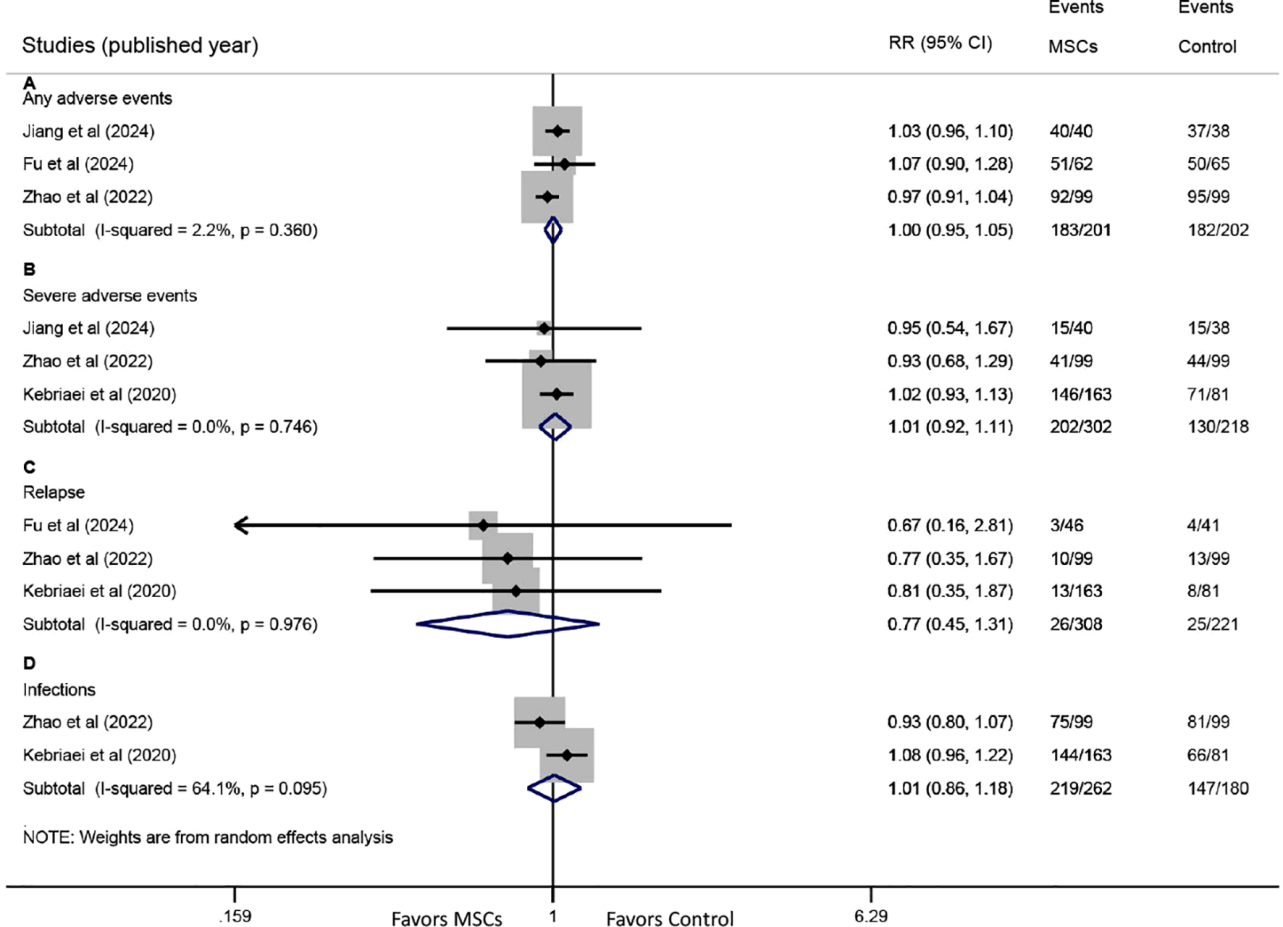
Forest plots for safety endpoints. Forest plots comparing the incidence of **(A)** any adverse events (AEs), **(B)** severe AEs, **(C)** primary disease relapse, and **(D)** infections between the MSC group and the control group.

Three studies reported data on any AEs. The pooled analysis showed that the incidence of any AEs was comparable between the MSC group and the control group (RR: 1.00, 95% CI: 0.95-1.05, P = 0.961) (**Figure 6A**). Similarly, three studies provided data on severe AEs, with meta-analysis indicating no significant difference between the groups (RR: 1.01, 95% CI: 0.92-1.11, P = 0.795) (**Figure 6B**).

Three studies reported relapse rates of the primary disease. Pooled results revealed no significant difference between the MSC and control groups (RR: 0.77, 95% CI: 0.45-1.31, P = 0.332) (**Figure 6C**). Data on infections were available from two studies, and no significant difference was observed between the two groups (RR: 1.01, 95% CI: 0.86-1.18, P = 0.922) (**Figure 6D**).

## 4 Discussion

This meta-analysis, based on data from four RCTs, demonstrates that the addition of MSCs to second-line therapy significantly improves both OR and CR rates in patients with steroid-refractory aGVHD following allo-HSCT. Furthermore, MSC treatment was associated with a significantly reduced incidence of chronic GVHD and prolonged failure-free survival, while maintaining a safety profile comparable with that of standard second-line therapy alone.

Unlike some rapid-acting second-line agents, MSCs exhibit a more gradual onset of action (23). This supports the rationale for combining MSCs with other second-line treatments—such as basiliximab or calcineurin inhibitors—to harness both the early benefits of conventional therapies and the sustained immunomodulatory effects of MSCs through complementary mechanisms. In our analysis, the combination of MSCs and second-line therapy achieved an OR rate of 71.4% (262/367) in steroid-refractory aGVHD, a finding consistent with a previous meta-analysis of non-randomized studies that reported an OR rate of 72% in comparable patient populations (27).

Our meta-analysis revealed significantly higher OR rates in patients with skin or gut involvement, as well as in those with multiorgan involvement, but not in patients with liver involvement or single-organ disease. One possible explanation is that multiorgan involvement reflects a more severe inflammatory state, which may respond more favorably to MSC therapy due to its dual mechanisms of immunomodulation and tissue repair (28, 29). It should also be noted that the number of patients with liver involvement included in this analysis was relatively small; therefore, these results should be interpreted with caution.

In contrast to previous studies suggesting that pediatric patients with steroid-refractory aGVHD may respond better to MSCs than adults (16, 30, 31), our meta-analysis showed a significant improvement in OR among adult patients, but not in pediatric subgroups. This discrepancy may be attributable to the limited number of pediatric patients included (49 in the MSC group and 44 in the control group), which likely underpowered the subgroup analysis to detect a significant treatment effect. Thus, the efficacy of MSCs in children warrants further investigation in larger, prospective studies. It is noteworthy that the study by Kurtzberg et al. (32), which supported the US FDA approval of MSC therapy for pediatric steroid-refractory aGVHD, reported an OR rate of 70.4% with Remestemcel-L treatment in children. This finding is consistent with the pooled OR rate of 71.4% observed in our meta-analysis.

MSCs derived from different sources exhibit distinct immunomodulatory properties and clinical benefits in GVHD (33, 34). This meta-analysis found that BM-MSCs were associated with a significantly higher OR, whereas UC-MSCs did not show a statistically significant effect. Two observations warrant attention: First, there was a trend toward improved OR in patients receiving UC-MSCs (P = 0.063); second, the two RCTs utilizing UC-MSCs had relatively small sample sizes compared with those using BM-MSCs (ranging from 78 to 260 patients across studies). This variability is further evidenced by results from pivotal clinical trials of commercially approved MSC products: Temcell (Japan-approved BM-MSCs) reported an OR of 61% (35), Remestemcel-L (US-approved BM-MSCs) achieved 70.4% in its pivotal trial (32), while Amimatoside Injection (China-approved UC-MSCs) demonstrated 71.9% in a phase II RCT (23). Given the heterogeneous outcomes across both investigational and approved products, the optimal MSC source warrants further investigation through well-designed randomized comparative trials.

Steroid-refractory aGVHD is a known risk factor for subsequent chronic GVHD (36). Our analysis suggests that the beneficial effects of MSCs in aGVHD extend to a reduced incidence of chronic GVHD and improved FFS. However, no significant improvement in OS was observed. This lack of OS benefit should be interpreted with caution due to the limited sample size and relatively short follow-up periods in the included studies.

This meta-analysis has several limitations that should be considered when interpreting the findings. First, the inclusion of only four RCTs with relatively small total sample sizes substantially limits the statistical power of our analysis, particularly in subgroup assessments. This constraint necessitates that conclusions regarding specific subpopulations—such as pediatric patients or those with liver involvement—be viewed as preliminary rather than definitive. Second, the clinical heterogeneity across the included studies must be acknowledged. Variations in MSC sources (UC versus BM), dosing regimens, and background second-line therapies likely influenced the outcomes and contributed to the observed inconsistencies, potentially affecting the generalizability of the pooled results. Finally, the efficacy of combining MSCs with newer agents like ruxolitinib (37, 38), which is now a standard therapy for steroid-refractory aGVHD, remains an important unanswered question and warrants investigation in future trials.

In summary, this meta-analysis indicates that the addition of MSCs to second-line therapy significantly improves overall and complete response rates in patients with steroid-refractory aGVHD and is associated with prolonged FFS and a comparable safety profile. These findings support the use of MSCs as an effective and safe therapeutic option for steroid-refractory aGVHD in patients undergoing allo-HSCT for hematological diseases.

## Data Availability

The original contributions presented in the study are included in the article/**Supplementary Material**. Further inquiries can be directed to the corresponding authors.
