# Supplementary material for: Mesenchymal stem cells added to second-line therapy improve response and failure-free survival in steroid-refractory acute graft-versus-host disease after allogeneic hematopoietic stem cell transplantation: A meta-analysis of randomized controlled trials
Source: Front Oncol. 2025 Nov 4;15:1704963. doi: 10.3389/fonc.2025.1704963 (PMC12623177; doi:10.3389/fonc.2025.1704963)
Supplement: Supplementary file 1 [file DataSheet1.docx]

**Table. S1 Search criterion of PubMed (from inception to** **May 10, 2025)**

| **No.** | **Query Results** | **Results** |
| --- | --- | --- |
| #23 | #14 AND #19 AND #22 | 89 |
| #22 | #20 OR #21 | 1922219 |
| #21 | Search: **"Randomized Controlled Trials as Topic"[Mesh] OR "Randomized Controlled Trial" [Publication Type]** Sort by: **Most Recent** | 829269 |
| #20 | Search: **random*[Text Word]** | 1920400 |
| #19 | #15 OR #16 OR #17 OR #18 | 45148 |
| #18 | Search: **"Graft vs Host Disease"[Mesh]** Sort by: **Most Recent** | 29516 |
| #17 | Search: **GVHD[Title/Abstract]** | 21425 |
| #16 | Search: **graft versus host[Title/Abstract]** | 31761 |
| #15 | Search: **graft vs host[Title/Abstract]** | 3043 |
| #14 | #1 OR #2 OR #3 OR #4 OR #5 OR #6 OR #7 OR #8 OR #9 OR #10 OR #11 OR #12 OR #13 | 102215 |
| #13 | Search: **"Mesenchymal Stem Cell Transplantation"[Mesh] OR "Mesenchymal Stem Cells"[Mesh]** Sort by: **Most Recent** | 62822 |
| #12 | Search: **MSCs[Title/Abstract]** | 40417 |
| #11 | Search: **adipose stem cells[Title/Abstract]** | 1363 |
| #10 | Search: **adipose stem cell[Title/Abstract]** | 464 |
| #9 | Search: **marrow stromal cells[Title/Abstract]** | 7291 |
| #8 | Search: **marrow stromal cell[Title/Abstract]** | 1735 |
| #7 | Search: **wharton's jelly[Title/Abstract]** | 1856 |
| #6 | Search: **mesenchymal precursor cells[Title/Abstract]** | 247 |
| #5 | Search: **mesenchymal precursor cell[Title/Abstract]** | 50 |
| #4 | Search: **mesenchymal stromal cells[Title/Abstract]** | 11171 |
| #3 | Search: **mesenchymal stromal cell[Title/Abstract]** | 2809 |
| #2 | Search: **mesenchymal stem cells[Title/Abstract]** | 64980 |
| #1 | Search: **mesenchymal stem cell[Title/Abstract]** | 18519 |

**Table. S2 Search criterion of Embase (from inception to May 10, 2025)**

| **No.** | **Query Results** | **Results** |
| --- | --- | --- |
| #23 | #14 AND #19 AND #22 | 361 |
| #22 | #20 OR #21 | 2814275 |
| #21 | 'randomized controlled trial (topic)'/exp | 300489 |
| #20 | random* | 2814170 |
| #19 | #15 OR #16 OR #17 OR #18 | 105883 |
| #18 | 'graft versus host disease'/exp | 93038 |
| #17 | gvhd:ab,ti | 49149 |
| #16 | ('graft'/exp OR graft) AND versus AND host:ab,ti | 59650 |
| #15 | ('graft'/exp OR graft) AND vs AND host:ab,ti | 13166 |
| #14 | #1 OR #2 OR #3 OR #4 OR #5 OR #6 OR #7 OR #8 OR #9 OR #10 OR #11 OR #12 OR #13 | 207369 |
| #13 | 'mesenchymal stem cell'/exp | 97786 |
| #12 | msc*:ab,ti | 56458 |
| #11 | adipose AND ('stem'/exp OR stem) AND cells:ab,ti | 31663 |
| #10 | adipose AND ('stem'/exp OR stem) AND cell:ab,ti | 24590 |
| #9 | ('marrow'/exp OR marrow) AND stromal AND cells:ab,ti | 36778 |
| #8 | ('marrow'/exp OR marrow) AND stromal AND cell:ab,ti | 30078 |
| #7 | wharton AND jelly:ab,ti | 2876 |
| #6 | mesenchymal AND ('precursor'/exp OR precursor) AND cells:ab,ti | 4418 |
| #5 | mesenchymal AND ('precursor'/exp OR precursor) AND cell:ab,ti | 3649 |
| #4 | mesenchymal AND stromal AND cells:ab,ti | 37237 |
| #3 | mesenchymal AND stromal AND cell:ab,ti | 30460 |
| #2 | mesenchymal AND ('stem'/exp OR stem) AND cells:ab,ti | 137783 |
| #1 | mesenchymal AND ('stem'/exp OR stem) AND cell:ab,ti | 111380 |

**Table. S3 Search criterion of Cochrane Library (from inception to May 10, 2025)**

| **No.** | **Query Results** | **Results** |
| --- | --- | --- |
| #1 | mesenchymal stem cell:ti,ab,kw | 2363 |
| #2 | mesenchymal stromal cell:ti,ab,kw | 600 |
| #3 | mesenchymal precursor cell:ti,ab,kw | 73 |
| #4 | wharton's jelly:ti,ab,kw | 113 |
| #5 | marrow stromal cell:ti,ab,kw | 320 |
| #6 | adipose stem cell:ti,ab,kw | 739 |
| #7 | MSC*:ti,ab,kw | 2488 |
| #8 | MeSH descriptor: [Mesenchymal Stromal Cells] explode all trees | 373 |
| #9 | #1 OR #2 OR #3 OR #4 OR #5 OR #6 OR #7 OR #8 | 4164 |
| #10 | graft vs host:ti,ab,kw | 1789 |
| #11 | graft versus host:ti,ab,kw | 2773 |
| #12 | GVHD:ti,ab,kw | 2488 |
| #13 | MeSH descriptor: [Graft vs Host Disease] explode all trees | 1055 |
| #14 | #10 OR #11 OR #12 OR #13 | 3542 |
| #15 | #9 AND #14 | 169 |


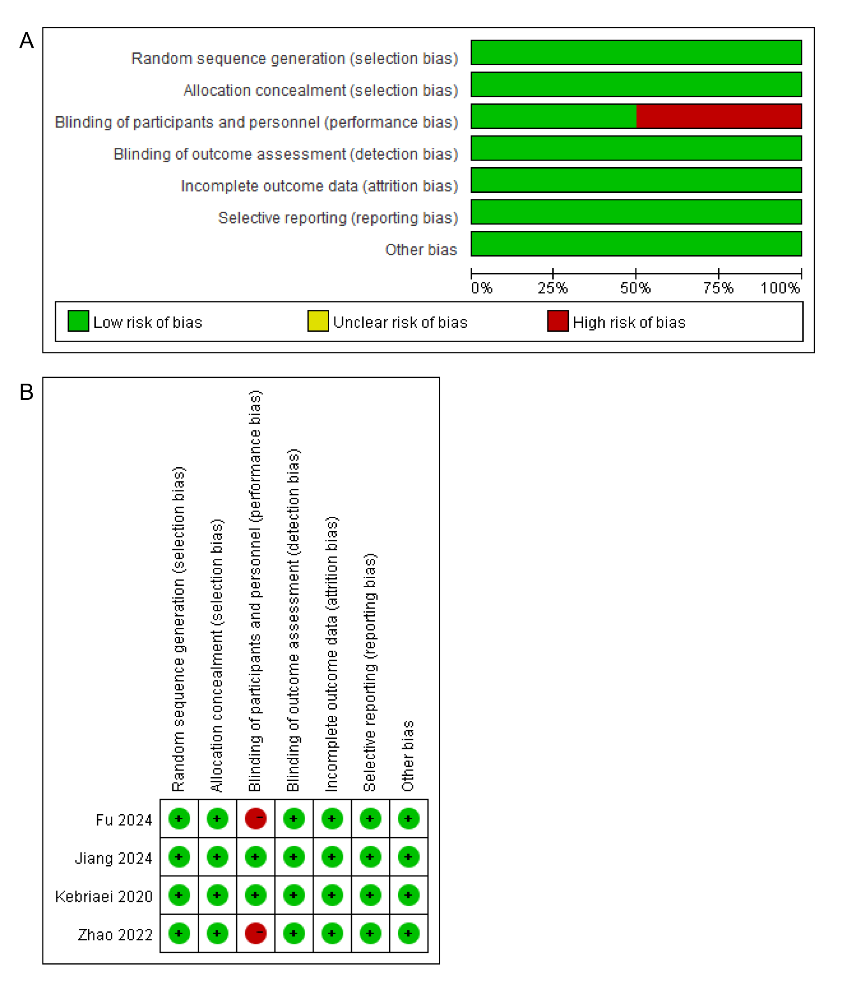


**Figure S1.** Methodological quality of the included studies. (**A**) Risk of bias graph. (**B**) Risk of bias summary. The minus sign indicates a high risk of bias, and the plus sign indicates a low risk of bias.
